# Single-Dose Therapy of Zoledronic Acid for the Treatment of Primary Bone Marrow Edema Syndrome

**DOI:** 10.7759/cureus.13977

**Published:** 2021-03-18

**Authors:** Angelo V Vasiliadis, Christianna Zidrou, George Charitoudis, Anastasios Beletsiotis

**Affiliations:** 1 2nd Orthopaedic Department, General Hospital of Thessaloniki “Papageorgiou”, Thessaloniki, GRC

**Keywords:** bone marrow edema, bisphosphonate, zoledronic acid, visual analogue scale, magnetic resonance imaging

## Abstract

Objective

To review the patients diagnosed with bone marrow edema syndrome who had been treated with one single dose of zoledronic acid.

Methods

The data of 54 patients with bone marrow edema syndrome treated with a single dose of intravenous zoledronic acid and partial weight-bearing were included in the study. The diagnosis was based on clinical examination, the existence of prolonged pain, the presence of bone marrow edema on magnetic resonance imaging, and the patient’s medical history. The efficacy was assessed using changes in symptoms, visual analogue scale, and changes in magnetic resonance imaging.

Results

Overall, 54 patients (35.2% male) were included with bone marrow edema syndrome, with a mean age of 52.7 ± 9.77 years (range: 35 - 74 years). The most commonly affected joint was the knee in 32 patients (59.2%), followed by the foot/ankle in 13 patients (24.1%), and the hip in nine patients (16.7%). Improved mobility was reported by 29 patients (53.7%) among the total number of the patients at the six-month follow-up visit. The mean visual analogue scale was 6.77 ± 0.83, 7.25 ± 1.19, and 7.46 ± 0.96 at baseline and 5.11 ± 2.14, 4.25 ± 1.84 and 5.15 ± 2.03 at the six-month follow-up for the hip, knee and foot/ankle, respectively (p = 0.098, p* *< 0.001, p* *= 0.002). At the six-month follow-up, the MRI showed resolution of the edema in 20 out of 54 patients (approximately 37%). Only 7.4% of the patients reported minor adverse events which were resolved through a single administration of paracetamol.

Conclusion

Our data show that the combination treatment of a single dose of zoledronic acid and partial weight-bearing for one month improves mobility and reduces edema in patients with bone marrow edema syndrome in the primary weight-bearing joints.

## Introduction

Bone marrow edema syndrome (BMES) refers to a clinical and radiological condition of uncertain etiology which is characterized by the accumulation of excessive volume of fluid in the related structures of bone marrow. Several hypotheses for the potential pathogenesis of BMES have been described [1-2]. The most recognized hypothesis suggests that it is caused by nerve compression or it is the result of an alteration in the venous outflow, causing increased intramedullary pressure [1]. However, new clinical and histological studies on bone metabolism in BMES proposed that vitamin D deficiency may play a negative role in bone mineralization to BMES [3-4]. Vitamin D deficiency leads to a decreased calcium absorption and, ultimately, the release of calcium from the bones in order to maintain circulating calcium concentrations. BMES mainly affects the hip, the knee, and the ankle joint of middle-aged males. The main symptom is pain in the affected bone with accompanying local tenderness, restricted range of motion (ROM), and inability to bear weight on the affected limb [5].

In terms of imaging modalities, magnetic resonance imaging (MRI) can clearly reveal a lower sequence signal intensity on T1-weighted scans, higher signal intensity on fat-suppressed T2-weighted scans, and short-tau inversion recovery (STIR) [1, 5]. Due to the heterogeneous and non-specific clinical presentation, diagnosis can be delayed, leading to poor functional outcomes with a devastating impact on the quality of life. Spontaneous recovery of symptoms has been reported in around six to 12 months, while other studies described incomplete remission of symptoms beyond 12 months [2, 6].

There is a controversy in the treatment of BMES. However, treatment with bisphosphonates has been described in recent years and has shown a significant reduction in pain and bone marrow edema size with normalization of MRI changes [1, 7-8]. It seems that bisphosphonates have a positive effect on bone metabolism by inhibiting osteoclasts and reducing bone resorption. Therefore, the role of bisphosphonates on bone remodeling and inflammatory conditions might be effective in reducing bone pain [2, 6]. Thus, the purpose of the present study was to evaluate the clinical and radiological outcome of patients diagnosed with BMES who were treated with a single dose of zoledronic acid. We hypothesize that zoledronic acid, combined with partial weight-bearing for a month, will improve joint pain over three and six months and reduce edema size over six months.

## Materials and methods

All patients who were diagnosed with BMES in our department between June 2019 and May 2020 were included. The present prospective study was examined and duly approved by our hospital’s Institutional Review Board. The diagnosis of BMES was based on clinical examination, the existence of prolonged hip, knee, and foot/ankle pain, the presence of abnormal bone marrow signal intensity in T1- and T2-weighted MRI, and the patient’s medical history. The exclusion criteria were (i) prior use of bisphosphonates, (ii) patients who had a corticosteroid or hyaluronic acid intra-articular injection, (iii) severe hip, knee, or ankle osteoarthritis, (iv) other forms of arthritis (e.g., rheumatoid arthritis or other inflammatory arthritis), (v) patients who had undergone arthroscopy or open surgery, (vi) patients who had planned joint replacement surgery, and (vii) patients with renal disorders. Informed consent was obtained from all the patients before starting the treatment.

The visual analogue scale (VAS) was noted before starting the treatment and at three- and six-month follow-up visits. The VAS consisted of a scale from 0 to 10, with 0 indicating no pain and 10 indicating the worst possible pain. The gait and the range of motion were assessed clinically before starting the treatment and at the three- and six-month follow-up visits. MRI scan of the affected joint was repeated at six months following treatment. Bone marrow edema was assessed by areas of decreased signal intensity on T1-weighted images and increased signal intensity on T2-weighted images.

As per standard practice followed in our department, all patients were administered the same treatment protocol and were given intravenous 100 ml of fluid containing zoledronic acid (Aclasta), 5 mg/100 ml (Novartis, Basel, Switzerland). All patients were also advised to practice partial weight-bearing for a month and then as tolerated. Any adverse effects of the intravenous zoledronic acid administration were also evaluated.

Collected data were analyzed with the Statistical Package for Social Sciences (SPSS), Version 24.0 (IBM SPSS Statistics for Windows, Armonk, NY). Continuous variables (age, body mass index (BMI), VAS) are expressed as mean, standard deviation (SD), and categorical variables (gender, smoking status, alcohol consumption, anatomic location, and clinical and radiological assessment) as percentages. The Kolmogorov-Smirnov test was utilized for normality analysis. The Mann-Whitney U-test and Kruskal Wallis test were utilized for the comparison of the continuous variables in our independent samples, for not normal distribution, in our population divided into two or more subpopulations, respectively. Pearson-χ2 (cross-tabulation) test was utilized for the comparison of the categorical variables. An analysis of variance (ANOVA) with repeated measures was used to compare the means of the continuous variables of the same three subpopulations, which were measured multiple times. The level of significance was set at p < 0.05.

## Results

**Figure 1:**
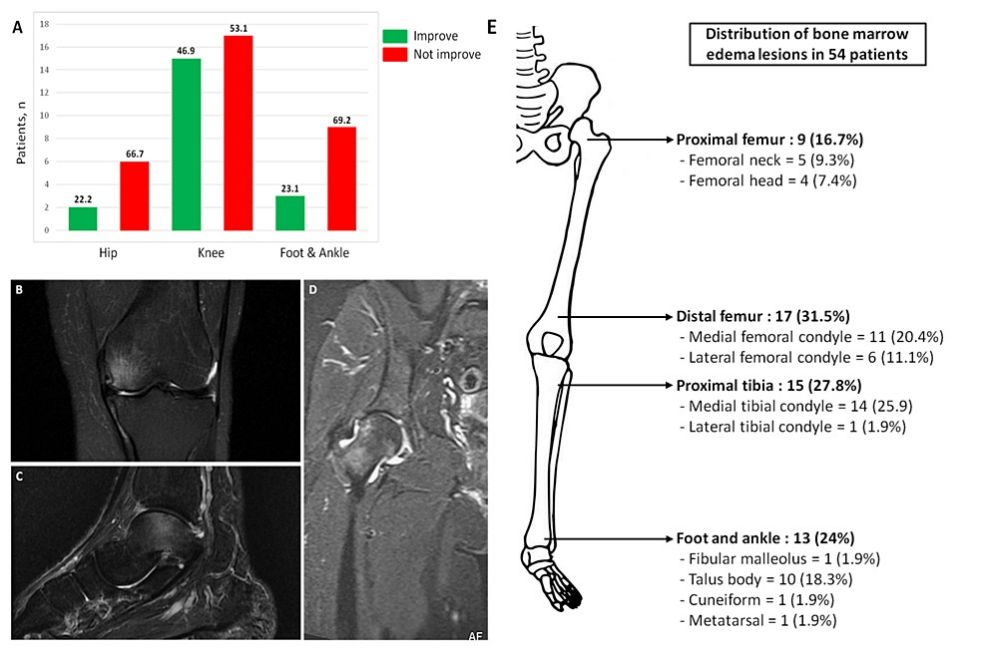
Distribution of the 54 patients with primary bone marrow edema syndrome (BMES) A) Radiological assessment with magnetic resonance imaging (MRI) of patients with BMES (numbers on the bar chart represent percentages); B-D) MRI scans of BMES in the medial femoral condyle of the knee, ankle (talus), and hip joints; E) anatomical location of the lesions

Data of 54 patients (19 males, 35.2%) with MRI confirmed BMES were analyzed (Figure 1). The mean age of the patients was 52.7 ± 9.77 years (range: 35 - 74 years). All patients had unilateral joint involvement of the hip, knee, or foot/ankle. The knee was the most frequently affected joint, with a total of 32 cases (59.3%). Fourteen patients (25.9%) were active smokers and six patients (11.1%) reported regular drinking. Analyzing the age of the patients, there was a statistically significant difference between patients with BMES in the knee and patients with BMES in the hip joint (p = 0.008), as well as patients with BMES in the foot/ankle and patients with BMES in the hip joint (p = 0.027). There were not any significant differences in relation to gender, BMI, smoking status, alcohol consumption, and comorbidities (Table 1).

**Table 1:** Demographic and Epidemiological Characteristics of Patients with Bone Marrow Edema Syndrome ^ significant at the level of < 0.05 between the hip and foot/ankle in accordance with the gender * significant at the level of < 0.01 between the knee and hip ** significant at the level of < 0.05 between the foot/ankle and hip # significant at the level of < 0.05 between the hip, knee, and foot/ankle BMI: body mass index; CVD: cardiovascular disease; GERD: gastroesophageal reflux disease; F/A: foot/ankle; M: male; NSSD: no statistically significant difference; SSD: statistically significant difference

	Total (n = 54)	Hip (n = 9)	Knee (n = 32)	F/A (n = 13)	p-value
Gender, M (%)	19 (35.2)	6 (66.7)^^^	11 (34.4)	2 (15.4)^^^	SSD
Age	52.7 ± 9.7	45.4 ± 4.6	54.5 ± 10.4*	53.2 ± 8.6**	SSD
BMI	28.9 ± 3.8	28.9 ± 4.9	29.6 ± 3.6	27.5 ± 3.1	NSSD
Smoking status					NSSD
None, n (%)	40 (74.1)	6 (66.7)	25 (78.1)	9 (69.2)	
Active, n (%)	14 (25.9)	3 (33.3)	7 (21.9)	4 (30.8)	
Alcohol consumption					NSSD
Yes, n (%)	6 (11.1)	3 (33.3)	2 (6.3)	1 (7.7)	
No, n (%)	48 (88.9)	6 (66.7)	30 (93.8)	12 (92.3)	
Co-morbidities					
All, n (%)	24 (44.4)	1 (11.1)	17 (53.1)	5 (38.5)	NSSD
Hypertension, n (%)	10 (18.5)	1 (11.1)	5 (15.6)	4 (30.8)	NSSD
CVD, n (%)	5 (9.3)		3 (9.4)	2 (15.4)	NSSD
Hypothyroidism, n (%)	8 (14.8)		8 (25)^ #^		SSD
Diabetes mellitus, n (%)	3 (5.6)		2 (6.3)	1 (7.7)	NSSD
Dyslipidemia, n (%)	8 (14.8)		8 (25)^ #^		SSD
Depression, n (%)	6 (11.1)	1 (11.1)	3 (9.4)	2 (15.4)	NSSD
GERD, n (%)	2 (3.7)		1 (3.1)	1 (7.7)	NSSD
Gout, n (%)	2 (3.7)		2 (6.3)		NSSD
Anatomic location					
Femoral head	5 (9.2)	5 (55.6)			
Femoral neck	4 (7.4)	4 (44.4)			
Medial femoral condyle	11 (20.4)		11 (34.4)		
Lateral femoral condyle	6 (11.1)		6 (18.8)		
Medial tibial condyle	14 (25.9)		14 (43.8)		
Lateral tibial condyle	1 (1.9)		1 (3.1)		
Talus body	10 (18.5)			10 (76.9)	
Fibular malleolus	1 (1.9)			1 (7.7)	
Cuneiform	1 (1.9)			1 (7.7)	
Metatarsal	1 (1.9)			1 (7.7)	

Forty-nine patients (90.7%) reported a limp in their gait and 24 patients (44.4%) experienced pain in the affected joint with active or passive range of motion without weight-bearing. The average VAS score before the beginning of the treatment was 6.77 ± 0.83, 7.25 ± 1.19, and 7.46 ± 0.96 for the hip, knee, and foot/ankle, respectively (Table 2). At the six-month follow-up, the limp had completely disappeared in 3, 18, and 3 patients with BMES in the hip, knee, and foot/ankle, respectively. No statistical significance was observed regarding the anatomical location of the hip and VAS score at six months follow-up (p = 0.098). However, statistical significance was observed regarding the knee, foot/ankle, and VAS score at the six-month follow-up (p < 0.001 and p = 0.002). Baseline and six-month follow-up MRIs were available in all of the patients. Out of these 54 patients, two patients with BMES in the hip, 15 patients with BMES in the knee, and three patients with BMES in the foot/ankle showed the disappearance of edema in the MRI (Figure 1). No major adverse effects were reported. Only four out of the 54 patients reported minor adverse events, such as fever (< 38 °C) and myalgia, which were resolved with the use of paracetamol.

**Table 2:** Clinical Assessment of Patients with Bone Marrow Edema Syndrome * significant at the level of < 0.001 between different time point follow-up ** significant at the level of < 0.05 between different time point follow-up NSSD: no statistically significant difference; ROM: range of motion; SSD: statistically significant difference; VAS: visual analogue score

	Baseline	3 months	6 months	p-value
Hip				
Gait - limp, n (%)	9 (100)	1 (11.1) *	6 (66.7)	SSD
ROM - painful, n (%)	8 (88.9)	0 (0) *	0 (0) *	SSD
VAS	6.77 ± 0.83	4.88 ± 0.78*	5.11 ± 2.14	SSD
Knee				
Gait - limp, n (%)	28 (87.5)	13 (40.6) *	10 (31.3) *	SSD
ROM - painful, n (%)	6 (18.8)	3 (9.4)	3 (9.4)	NSSD
VAS	7.25 ± 1.19	5.03 ± 1.55*	4.25 ± 1.84*	SSD
Foot/ankle				
Gait - limp, n (%)	12 (92.3)	3 (23.1) *	9 (69.2)	SSD
ROM - painful, n (%)	10 (76.9)	8 (61.5)	1 (7.7) *	SSD
VAS	7.46 ± 0.96	5.53 ± 1.26**	5.15 ± 2.03*	SSD

## Discussion

In our prospective study, we found that a single dose of intravenous zoledronic acid, combined with partial weight-bearing, provided a good response for both clinically and radiographic outcomes in patients with BMES [5, 7, 9]. Numerous studies have assessed the clinical and radiological characteristics of the BMES. They showed a clear predominance of male patients [5, 7, 9-10], and the vast majority of the lesions were described in the lower extremities, with the proximal site of the femur being the most common anatomical location, followed by the knee and the foot/ankle [2, 10-12]. In contrast, in our study, we found a female predominance with a male-to-female ratio of 1:1.84. Moreover, unlike previously published studies, the lesions in our study were noticeably concentrated in the knee, followed by the foot/ankle and the proximal femur (head and neck). A possible explanation is the fact that previous studies may focus on a selected anatomical region (or joint) [6, 11-12]. Our results are supported by the finding of Cahir and Toms who evaluated multiple anatomical regions and found that the knee was the most affected joint [13].

Prior research has demonstrated that the mean age of disease onset in BMES is between 50 and 70 years [1, 3, 6]. In our study, a large peak of BMES in patients aged 50 - 60 years was also demonstrated, but there was a second smaller peak of BMES in female patients aged below 45 years. This interesting observation possibly reflects the fact that perimenopause was associated with relatively insufficient bone mineralization, which can increase the risk of developing osteoporosis and bone fracture [14].

To date, studies carried out using bisphosphonates on BMES are mostly retrospective. Numerous bisphosphonates, such as zoledronic acid, alendronate, or ibandronate, have been used in a small number of patients [2, 6-7, 9]. The present prospective study was carried out using a single dose of zoledronic acid in patients diagnosed with BMES in three anatomical regions (hip, knee, and foot/ankle). Our results are similar to those from previously published studies done with other bisphosphonates [6-7, 9]. Zoledronic acid has the benefit of being administrated intravenously once per year, has a low cost, and has no severe adverse effects.

Several studies have shown that although zoledronic acid is effective in completely reversing the clinical process of the disease, its efficacy may be observed late (from four weeks for the reduction of the pain and 14 weeks to 12 months for the complete resolution of pain) [2, 6, 9]. In a study by Muller et al., 12 patients with symptomatic bone marrow lesion of the knee were treated with intravenous zoledronic acid once only, and a complete resolution of the symptoms was observed in 11 patients (92%) four weeks after the application of the medication [9]. Similarly, a single dose of 5 mg of zoledronic acid in 17 patients with primary BMES in the ankle showed pain reduction in 15 patients (88%) at three months and complete disappearance of the pain in 13 patients (76.4%) after 12 months [6].

Although intravenous administration of bisphosphonates alone has shown promising results, combination therapies with ibandronate and vitamin D have revealed excellent pain reduction (at rest and under strain) and improved mobility within the first two weeks of administration in high-performance athletes [7]. In addition, Singh et al. demonstrated that protected weight-bearing coupled with intravenous administration of zoledronic acid in patients with BMES of the foot/ankle appeared to speed up the time to recovery in their series when compared with protected weight-bearing alone [2]. Our study has shown that intravenous zoledronic acid, plus partial weight-bearing for a month, can reduce the pain and be effective in two-thirds of the patients with BMES in the knee and in one-third of the patients with BMES in the hip and foot/ankle.

It should be noted that the patients in our study showed a limited normalization or/and reduction of the initial edema on MRI in only 20 patients (37%) in total, with the knee joint alone showing the best results in 15 out of 32 patients (46.9%). In contrast, Flores-Robles et al. revealed that from the available MRIs in the follow-up period (three months after the treatment administration) edema had disappeared in 62.5% of the patients, while a reduction of the initial edema more than 50% was observed in three patients (37.5%) [6]. In accordance with the previous study, Simon et al. demonstrated a significant reduction or even complete regression of bone marrow edema after bisphosphonates administration with a mean follow-up of 13 months [7]. A possible explanation for this is the fact that normalization of MRI findings occurred around three to 18 months after they appeared [6, 8].

## Conclusions

The present study shows that a single dose of intravenous zoledronic acid combined with partial weight-bearing for a month provides a good response for both clinically and radiographically outcomes in patients with BMES. These results seem to be better in patients with BMES in the knee compared with those of the hip and foot/ankle. Further prospective, randomized, and controlled trials with a longer follow-up are needed in order to confirm the beneficial effects of intravenous bisphosphonates treatment.
